# Gray Matter Volume of Thalamic Nuclei in Traumatized North Korean Refugees

**DOI:** 10.3389/fpsyt.2022.756202

**Published:** 2022-04-28

**Authors:** Jiye Lee, Nambeom Kim, Hyunwoo Jeong, Jin Yong Jun, So Young Yoo, So Hee Lee, Jooyoung Lee, Yu Jin Lee, Seog Ju Kim

**Affiliations:** ^1^Department of Psychiatry, Samsung Medical Center, Sungkyunkwan University School of Medicine, Seoul, South Korea; ^2^Neuroscience Research Institute, Gachon University, Incheon, South Korea; ^3^Geumsan-gun Public Health Center, Seoul, South Korea; ^4^Department of Psychiatry, Seoul National Hospital, Seoul, South Korea; ^5^Department of Psychiatry, National Medical Center, Seoul, South Korea; ^6^Department of Psychiatry and Center for Sleep and Chronobiology, Seoul National University Hospital, Seoul, South Korea

**Keywords:** thalamus, refugees, depression, trauma, magnetic resonance imaging

## Abstract

The current study investigated differences in the regional gray matter (GM) volume of specific thalamic nuclei between North Korean (NK) refugees and South Korean (SK) residents. It also investigated associations between thalamic GM volume changes and psychological symptoms. Psychological evaluations and magnetic resonance imaging were conducted on 50 traumatized NK refugees and 55 non-traumatized SK residents. The regional GM volume ratios in the bilateral thalami were calculated for all participants using voxel-based morphometry. NK refugees showed greater GM volume ratios in the right medial-posterior nuclei and left medial nuclei compared with SK residents. NK refugees also exhibited more depressive symptoms than SK residents. However, increased GM volume ratios in both right medial-posterior nuclei and left medial nuclei were correlated with fewer depressive symptoms in NK refugees, but not in SK residents. The findings indicate that traumatized NK refugees had increased GM volumes in the right medial-posterior nuclei and left medial nuclei, which were associated with fewer depressive symptoms. The enlarged specific thalamic nuclei presented among refugees in the current study might be associated with a neurobiological compensatory mechanism that prevents the development or progression of depression in refugees after repetitive traumatic experiences.

## Introduction

The thalamus has been presumed to play a pivotal role in fear processing, which is crucial for understanding trauma-related disorders [e.g., posttraumatic stress disorder (PTSD)] (1, 2). The hippocampal-prefrontal-thalamic circuitry functions in contextual fear conditioning; its disruption is presumed to be a core component of PTSD pathophysiology (2). The thalamus regulates fear learning and expression in the central amygdala by modulating the functions of neurons through the activities of brain-derived neurotrophic factor receptor tropomyosin-related kinase B (3). Alternating bilateral sensory stimulation reportedly led to long-lasting fear attenuation via thalamic activation in mice (4).

Trauma itself is known to affect brain structure and function regardless of PTSD development (5, 6). These brain changes include the change of thalamus after trauma (5). In addition, trauma can induce non-PTSD psychiatric symptoms, which are also related to brain structure and function (7–13). Therefore, we intended to explore the effects of trauma itself on thalamus.

Several previous studies have shown structural changes in the thalamus after trauma or severe stress (6, 14). Changes in thalamic volume have been observed in victims of childhood maltreatment or earthquakes (15, 16). The thalamus consists of various nuclei, each of which has a distinct location and function. Many thalamic nuclei are involved in fear conditioning and emotional regulation, which may be related to structural or functional changes of the thalamus after traumatic experiences (17–19). To our knowledge, no study has specifically investigated changes in the thalamic nuclei of traumatized people. In addition, the functional meaning of the structural changes in specific thalamic nuclei has not been reported. In the present study, we investigated which thalamic nuclei exhibit volumetric alterations in traumatized refugees and the clinical implications.

Among traumatized victims, refugees are an important population for examining the effect of chronic, repetitive, and severe trauma on brain structures such as the thalamus. Most refugees fled their communities because of war, civil conflict, disaster, oppression, or persecution (20). They may have experienced imprisonment, torture, physical assault, rape, and forced separation from family prior to fleeing (21, 22). Although brain structures and functions have been widely studied in other traumatized populations, only a few studies have been performed on alterations of brain structure in traumatized refugees. North Korean (NK) refugees reportedly experience chronic and repetitive trauma after defection from NK, prior to their arrival in South Korea (23–25). Structural and functional brain changes in NK refugees have been characterized in previous studies (26–28).

In this study, we compared regional gray matter volumes (GM volumes) in thalamic nuclei between NK refugees and South Korean (SK) residents. This study also explored associations between structural changes in thalamic nuclei and common psychological symptoms in traumatized people (e.g., depression). Based on previous studies, we hypothesized that GM volumes in specific thalamic nuclei would differ between NK refugees and SK residents. Additionally, we hypothesized that changes in thalamic GM volumes in NK refugees would be correlated with their psychological symptoms.

## Materials and Methods

### Participants

North Korean refugees staying in South Korea and native SK residents were recruited through advertisements. Participants with the following conditions were excluded: history of head injury, neurological disorder, untreated serious medical illness, and/or neurodevelopmental disorder, and any metal or electronic implants that violated magnetic resonance imaging (MRI) safety standards. The presence of a psychiatric disorder was an additional exclusion criterion for SK residents but not NK refugees. Among the initially recruited participants, three NK refugees were excluded because of structural abnormalities in the brain; one NK refugee was excluded because of poor image quality. Finally, 50 NK refugees (11 men and 39 women; mean age, 35.96 ± 11.05 years) and 55 SK residents (19 men and 36 women; mean age, 35.96 ± 11.07 years) participated in the study. All NK refugees had at least one traumatic experience, as defined by the Diagnostic and Statistical Manual for Mental Disorders, Fourth Edition (DSM-IV) criteria. Six NK refugees used psychotropic medications including antidepressants and sedatives. The psychiatric disorders of the NK refugees are shown in Table 1. Twenty-three NK refugees were diagnosed with current Axis I psychiatric disorders, according to the Structured Clinical Interview for DSM-IV (SCID-IV). Among these 23 NK refugees, the psychiatric disorders were as follows (some refugees had multiple disorders): PTSD (*n* = 3), depressive disorders (*n* = 11), anxiety disorders (*n* = 7), adjustment disorders (*n* = 3), somatoform disorders (*n* = 6), and eating disorders (*n* = 1). This study was approved by the Institutional Review Board of Seoul National University Hospital; all participants provided written informed consent before inclusion in the study.

**TABLE 1:** Demographic and clinical characteristics of North Korean (NK) refugees and South Korean (SK) residents.

Variables	NK refugees (*n* = 50)	SK residents (*n* = 55)	Group difference	
	Means ± SD or n (%)	Means ± SD or n (%)	T/χ^2^	*p*-value
Age (years)	35.96 ± 11.05	35.96 ± 11.07	−0.002	1.00
Number of females	39 (78.00%)	36 (65.45%)	2.02	0.16
Information regarding defection				
Time since defection (months)	114.60 ± 63.58	–	NA	NA
Length of stay in transit countries (months)	45.97 ± 51.53	–	NA	NA
Duration of habitation in South Korea (months)	64.84 ± 33.03	–	NA	NA
Number of traumatic experiences	4.91 ± 3.07	–	NA	NA
Psychiatric disorders		–		
Posttraumatic stress disorder	3 (6%)	–	NA	NA
Depressive disorder	11 (22%)	–	NA	NA
Anxiety disorder	7 (14%)	–	NA	NA
Adjustment disorder	3 (6%)	–	NA	NA
Somatoform disorder	6 (12%)	–	NA	NA
Eating disorder	1 (2%)	–	NA	NA
All psychiatric disorders	23 (46%)	–	NA	NA
Psychological questionnaire scores				
BAI***	19.98 ± 14.36	7.02 ± 7.61	5.29	<0.001
BDI**	16.18 ± 14.04	8.33 ± 8.63	3.27	0.002
IES-R	24.88 ± 21.17	–	NA	NA

*p < 0.05, **p < 0.01, ***p < 0.001.

*NK, North Korean; SK, South Korean; SD, standard deviation; BAI, Beck Anxiety Inventory; BDI, Beck Depression Inventory; IES-R, Impact of Event Scale-Revised.*

### Evaluation for Psychological Symptoms

All participants were assessed using the Korean version of the SCID-IV to diagnose psychiatric disorders. Korean versions of the Beck Anxiety Inventory (BAI) and Beck Depression Inventory (BDI) were administered to each participant to estimate the severity of anxiety and depression, respectively. Using the Impact of Event Scale-Revised (IES-R), the NK refugees were evaluated in terms of symptom severity in three PTSD domains (intrusion, avoidance, and hyperarousal). The Korean versions of the BAI (Cronbach α = 0.91) (29), BDI (Cronbach α = 0.89) (30), and IES-R (Cronbach α = 0.93) (31) have demonstrated excellent internal consistency and strong correlations with other inventories of anxiety, depression, and PTSD, respectively. The significant anxiety can be suspected when BAI scores were over 21 (32). According to the BDI, 14 is the cut-off point for significant depressive symptoms (30). The presence of PTSD symptoms was defined as IES-*R*≥24 (31).

The Trauma Exposure Check List for North Korean Refugees was used to evaluate the types and numbers of traumatic experiences reported by NK refugees (33). The checklist consists of 13 types of traumatic events that could be experienced in North Korea, as well as 16 types of traumatic events that can occur during defection to South Korea. These events include torture, severe battery, life-threatening starvation/cold/accidents, rape, human trafficking, arrest, imprisonment, and observation of violent death. The period of time after defection, length of stay in transit countries, duration of habitation in South Korea, and use of psychotropic medication were also assessed in interviews with the refugees.

### Magnetic Resonance Imaging Data Acquisition

Structural MRI data were acquired using a 3T MRI system (Trio Tim, Siemens; Erlangen, Germany) with a 12-channel birdcage head coil. Sagittal T1-weighted, 3D magnetization-prepared rapid gradient echo imaging was performed with the following parameters: repetition time = 1,670 ms, echo time = 1.89 ms, inversion time = 900 ms, flip angle = 9°, slice thickness = 1.0 mm, in-plane resolution = 1.0 mm × 1.0 mm, field of view = 250 mm, and matrix size = 256 × 256.

### Voxel-Based Morphometry

Voxel-based morphometry was conducted using the Diffeomorphic Anatomical Registration Through Exponentiated Lie (DARTEL) tool in SPM12.^1^ First, T1-weighted images were segmented (unified segmentation) into probability maps of gray and white matter, cerebrospinal fluid, skull and soft tissue outside the brain. A population gray matter template was created by high-dimensional non-linear warping, by applying DARTEL algebra to the probability maps of gray and white matter. Then, the gray matter probability maps of each participant were normalized to the population template. The population template was normalized to Montreal Neurological Institute (MNI) space using affine transformation; all individual gray matter maps normalized to the template space were then transformed into MNI space using the same transformation parameter with Jacobian modulation. Finally, images that had been normalized to MNI space were smoothed with a Gaussian kernel of 8-mm full-width at half-maximum (34).

Bilateral thalamic regions of interest were generated using the AAL template of Marsbar.^2^ GM volume in the thalamus was calculated, with small volume correction (SVC) done in SPM12.

Since VBM based methods may have low sensitivity to small structures and probability of misidentification of the nuclei, we conducted direct thalamic nuclei segmentation using Freesurfer (35). To identify areas with significant groupwise differences in GM volume, thalamic nuclei were separated and defined in accordance with the atlas in Freesurfer. The segmentation of thalamic nuclei was done after completing cortical segmentation (e.g., recon-all). The atlas consisted of 26 human thalamic nuclei based on *ex vivo* MRI and histology. It segmented the thalamic nuclei into anterior group (anteroventral nucleus), lateral group (laterodorsal, lateral posterior), ventral group (ventral anterior, ventral anterior magnocellular, ventral lateral anterior, ventral lateral posterior, ventral posterolateral, ventromedial), intralaminar group (central medial, central lateral, paracentral, centromedian, parafasciculuar), medial group (paratenial, reuniens [medial ventral], mediodorsal medial magnocellular, mediodorsal lateral parvocellular), and posterior group (lateral geniculate, medial geniculate, limitans [suprageniculate], pulvinar anterior, pulvinar medial, pulvinar lateral, pulvinar inferior). The volume of thalamic nuclei between NK refugees and SK residents was statistically tested using independent two-sample *t*-test. For controlling for multiple comparison, additional analysis with Bonferroni correction was also conducted.

### Data Analyses

To evaluate groupwise differences in thalamic GM volume, two-sample *t*-tests of bilateral thalami were conducted with SVC. Age, sex, and intracranial content volumes were entered as nuisance covariates. The significant level was set to a familywise error rate-corrected cluster level of *p* < 0.05, and an uncorrected peak level of *p* < 0.001. To further evaluate potential associations between clinical variables (e.g., BAI, BDI, and IES-R) and regional GM volumes in thalamic nuclei after the identification of significant groupwise differences, two-sample *t*-tests were used.

To compare the demographic and clinical data between the two groups, independent *t*-tests were used to analyze continuous variables and chi-squared tests to analyze categorical variables. Partial correlation analyses, adjusted for age, sex, and the number of traumatic experiences, were performed within each study group to identify associations between GM volume in the identified cluster and each clinical variable. These analyses were repeated with additional adjustment for psychotropic medication and psychiatric disorders. All statistical analyses were performed using R software (R Development Core Team, Vienna, Austria) and *p*-values < 0.05 were considered to indicate statistical significance.

In the additional analysis with direct thalamic nuclei segmentation, ANCOVA was used to compare GM volume ratios of thalamic nuclei adjusted for intracranial volume between the two groups. Age, sex, psychotropic medication, and psychiatric disorders were defined as covariates.

## Results

### Demographic and Clinical Characteristics

The demographic and clinical characteristics of the participants are shown in Table 1. The mean age did not significantly differ between NK refugees (35.96 ± 11.05 years) and SK residents (35.96 ± 11.07 years; *t* = −0.002, *p* = 1.00). Additionally, there were no significant differences in the sex ratio between NK refugees (39 women, 78%) and SK residents (36 women, 65.45%; chi = 2.02, *p* = 0.16).

The BAI and BDI scores were higher in NK refugees (BAI = 19.98 ± 14.36; BDI = 16.18 ± 14.04) than SK residents (BAI = 7.02 ± 7.61, *t* = 5.29, *p* < 0.001; BDI = 8.33 ± 8.63, *t* = 3.27, *p* = 0.002, respectively). Following adjustment for age and sex, NK refugees still showed significantly higher BAI and BDI scores compared with SK residents (BAI: *t* = 6.09, p < 0.001; BDI: *t* = 3.74, *p* < 0.001).

All NK refugees had at least one traumatic experience. The mean number of traumatic experiences was 4.91 ± 3.07. The mean interval after defection was 114.60 ± 63.58 months. The mean length of stay in transit countries (neither North Korea nor South Korea) was 45.97 ± 51.53 months. The mean duration of habitation in South Korea was 64.84 ± 33.03 months. The mean IES-R score of the NK refugees was 24.88 ± 21.17.

### Comparison of Thalamic Gray Matter Volume Ratio Between North Korean Refugees and South Korean Residents

**FIGURE 1:**
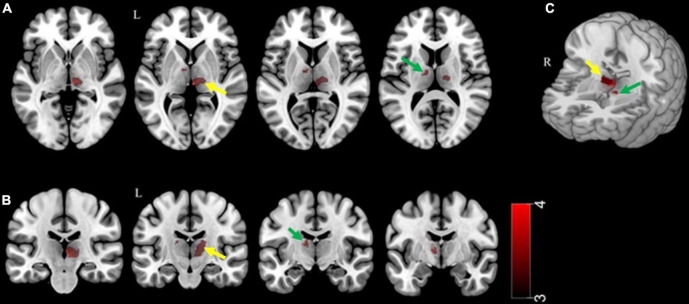
Thalamic areas showing increased gray matter volume in NK refugees compared with SK residents. Thalamic areas showing increased gray matter volume in NK refugees, compared with SK residents, are shown in the **(A)** axial plane, **(B)** coronal plane, and **(C)** 3D rendering. These areas correspond to the left medial nuclei (green arrow) and right medial-posterior nuclei (yellow arrow). The voxel-wise uncorrected statistical threshold was set at *p* < 0.001, and the cluster-wise familywise error rate-corrected threshold at *p* < 0.05, with small volume correction. NK, North Korean; SK, South Korean.

Compared with SK residents, NK refugees showed greater thalamic GM volumes in the right medial-posterior nuclei (*x* = 5, *y* = −23, *z* = 2, cluster size = 546, *Z*-score = 3.61) and left medial nuclei (*x* = −9, *y* = −6, *z* = 5, cluster size = 132, *Z*-score = 3.69) (Table 2). As the significant cluster overlied both parts of right medial and posterior nuclei, we designated the cluster as right medial-posterior nuclei. Figure 1 shows the brain areas with increased thalamic GM volume in the NK refugees compared with SK residents. The difference in cluster volume ratio remained statistically significant after adjustment for age and sex (right medial-posterior nuclei: *t* = 8.75, *p* < 0.001; left medial nuclei: *t* = 9.57, *p* < 0.001, respectively). After additional adjustment for psychotropic medication and psychiatric disorders, the GM volume ratios of the above-mentioned clusters remained greater in NK refugees than SK residents (right medial-posterior nuclei: *t* = 8.04, *p* < 0.001; left medial nuclei: *t* = 8.33, *p* < 0.001).

**TABLE 2:** Coordinates of thalamic areas showing increased gray matter volume in NK refugees compared with SK residents.

Cortical regions	MNI coordinates (mm) (x, y, z)			Cluster size	*Z*-scores
Right medial-posterior nuclei	5	−23	2	546	3.61
Left medial nuclei	−9	−6	5	132	3.69

*The voxel-wise uncorrected statistical threshold was set at p < 0.001, and the cluster-wise familywise error rate-corrected threshold at p < 0.05, with small volume correction.*

*NK, North Korean; SK, South Korean; MNI, Montreal Neurological Institute.*

Additional analysis was conducted to investigate the associations between GM volumes and psychiatric disorders in NK refugees. There were no significant differences in GM volumes in the right medial-posterior nuclei and left medial nuclei between NK refugees with and without psychiatric disorders after adjustment for age and sex. Additionally, in order to investigate the associations between GM volumes and psychiatric symptoms, we compared GM volumes in the clusters between NK refugees with and without psychiatric symptoms. There were no significant differences in GM volumes in the right medial-posterior nuclei and left medial nuclei between NK refugees with and without anxiety, depression or PTSD symptoms.

In the additional analysis with false discovery rate (FDR) corrected *p*-value, compared with SK residents, NK refugees showed greater GM volumes in the right medial-posterior nuclei (*x* = 5, *y* = −23, *z* = 2, cluster size = 546, *Z*-score = 3.61). In whole brain analysis with same threshold, NK refugees showed greater GM volumes than SK residents in the left middle frontal lobe (*x* = −45, *y* = 6, *z* = 51, cluster size = 1,394, *Z*-score = 4.2). Although left middle frontal GM volume was significantly associated with GM volumes of right medial-posterior nuclei (*r* = 0.56, *p* < 0.001) and left medial nuclei (*r* = 0.49, *p* < 0.001) in all participants, this significance disappeared after controlling the NK-SK group effects.

**FIGURE 2:**
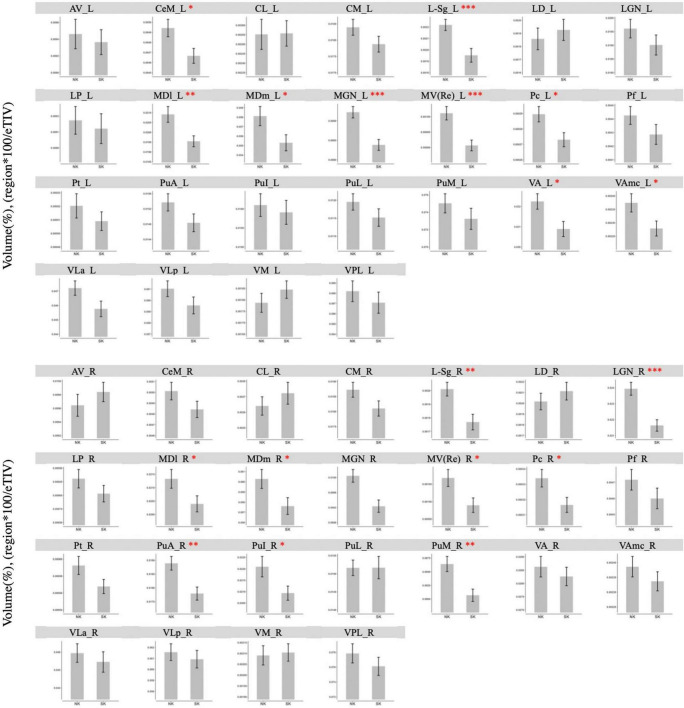
Comparison of gray matter volume of thalamic nuclei between NK refugees and SK residents in additional analysis using Freesurfer. In the direct thalamic nuclei segmentation using Freesurfer, NK refugees showed greater GM volumes in the left central medial, suprageniculate, mediodorsal lateral parvocellular, mediodorsal medial magnocellular, medial geniculate, reuniens, paracentral, ventral anterior, ventral anterior magnocellular nucleus and right suprageniculate, lateral geniculate, mediodorsal lateral parvocellular, mediodorsal medial magnocellular, medial geniculate, reuniens, paracentral, pulvinar anterior, pulvinar inferior, pulvinar medial nucleus than SK residents. *p < 0.05, ^**^p < 0.01, ^***^p < 0.001. AV, Anteroventral; LD, Laterodorsal; LP, Lateral posterior; VA, Ventral anterior; VAmc, Ventral anterior magnocellular; VLa, Ventral lateral anterior; VLp, Ventral lateral posterior; VPL, Ventral posterolateral; VM, Ventromedial; CeM, Central medial; CL, Central lateral; Pc, Paracentral; CM, Centromedian; Pf, Parafascicular; Pt, Paratenial; MV-re, Reuniens (medial ventral); MDm, Mediodorsal medial magnocellular; MDl, Mediodorsal lateral parvocellular; LGN, Lateral geniculate; MGN, Medial Geniculate; L-sg, Limitans (suprageniculate); PuA, Pulvinar anterior; PuM, Pulvinar medial; PuL, Pulvinar lateral; PuI: Pulvinar inferior.

In the direct thalamic nuclei segmentation using Freesurfer, NK refugees showed greater GM volumes in the left central medial (*p* = 0.02), suprageniculate (*p* < 0.001), mediodorsal lateral parvocellular (*p* = 0.006), mediodorsal medial magnocellular (*p* = 0.03), medial geniculate (*p* < 0.001), reuniens (*p* < 0.001), paracentral (*p* = 0.02), ventral anterior (*p* = 0.01), ventral anterior magnocellular nucleus (*p* = 0.03) and right suprageniculate (*p* = 0.002), lateral geniculate (*p* < 0.001), mediodorsal lateral parvocellular (*p* = 0.046), mediodorsal medial magnocellular (*p* = 0.04), medial geniculate (*p* < 0.001), reuniens (*p* = 0.02), paracentral (*p* = 0.02), pulvinar anterior (*p* = 0.002), pulvinar inferior (*p* = 0.04), pulvinar medial nucleus (*p* = 0.002) after controlling ICV than SK residents (Figure 2). After adjustment for age, sex, psychotropic medication and psychiatric disorders, the differences in GM volumes in the left central medial (*p* = 0.004), suprageniculate (*p* < 0.001), mediodorsal lateral parvocellular (*p* = 0.007), mediodorsal medial magnocellular (*p* = 0.02), medial geniculate (*p* < 0.001), reuniens (*p* < 0.001), paracentral (*p* = 0.003), ventral anterior (*p* < 0.001), ventral anterior magnocellular nucleus (*p* = 0.002) and right suprageniculate (*p* = 0.01), lateral geniculate (*p* < 0.001), mediodorsal lateral parvocellular (*p* = 0.04), mediodorsal medial magnocellular (*p* = 0.03), medial geniculate (*p* = 0.002), reuniens (*p* = 0.007), paracentral (*p* = 0.002), pulvinar anterior (*p* = 0.02), pulvinar inferior (*p* = 0.04), pulvinar medial nucleus (*p* = 0.009) remained statistically significant.

**FIGURE 3:**
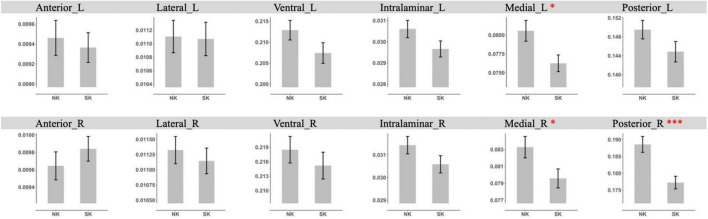
Comparison of gray matter volume of thalamic nuclei group between NK refugees and SK residents in additional analysis using Freesurfer. NK refugees showed greater GM volumes in the left medial group and right medial, posterior than SK residents. *p < 0.05, ^**^p < 0.01, ^***^p < 0.001.

In the additional analysis with grouping nuclei into anterior, lateral, ventral, intralaminar, medial, and posterior groups of nuclei, NK refugees showed greater GM volumes in the left medial group (*p* = 0.02) and right medial (*p* = 0.03), posterior group (*p* < 0.001) after controlling ICV than SK residents (Figure 3). After additional adjustment for age, sex, psychotropic medication and psychiatric disorders, the differences in GM volumes in the left medial group (*p* = 0.01) and right medial (*p* = 0.02), posterior group (*p* < 0.001) remained statistically significant. With Bonferroni corrected *p*-value, NK refugees showed greater GM volumes in the posterior group (p = 0.006) after controlling ICV, age, sex, psychotropic medication, and psychiatric disorders than SK residents. There were no significant between group differences in the left medial group (*p* = 0.06) and right medial (*p* = 0.10) with Bonferroni correction.”

### Relationships Between Thalamic Gray Matter Volume Ratios and Clinical Variables

**FIGURE 4:**
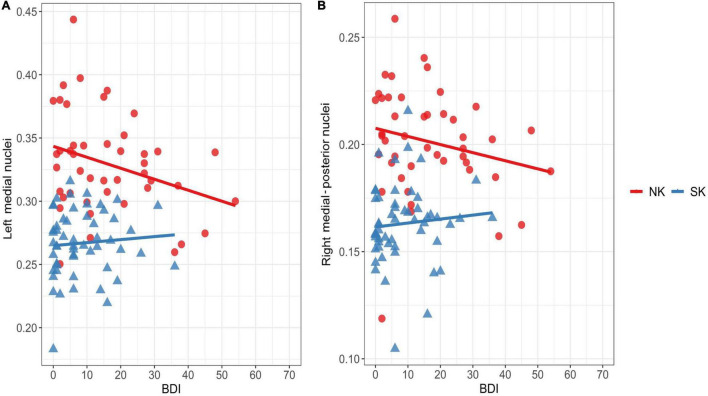
Correlations between depressive symptoms and thalamic areas showing increased gray matter volume in the NK refugees (red) SK residents (blue). Correlations are shown between the BDI score and gray matter volume ratio in the left medial nuclei **(A)** and right medial-posterior nuclei **(B)** is shown above. In the NK refugees alone, gray matter volume ratios in the left medial nuclei (*r* = –0.43, *p* = 0.009) and right medial-posterior nuclei (*r* = –0.33, *p* = 0.049) were negatively correlated with the BDI score after adjustment for age, sex, and the number of traumatic experiences. NK, North Korean; SK, South Korean; BDI, Beck Depression Inventory.

In the NK refugees, the GM volume ratio in the left medial nuclei was negatively correlated with the BDI score after adjustment for age, sex and the number of traumatic experiences [*r* = −0.43, *p* = 0.009) (Figure 4A)]. This correlation between the GM volume ratio in the left medial nuclei and the BDI score remained statistically significant despite additional adjustment for psychotropic medication and psychiatric disorders (*r* = −0.43, *p* = 0.02). Moreover, the GM volume ratio in the right medial-posterior nuclei was significantly correlated with the BDI score after adjustment for age, sex, and the number of traumatic experiences (*r* = −0.33, *p* = 0.049) (Figure 4B)]. However, this correlation between the GM volume ratio in the right medial-posterior nuclei and the BDI score did not remain statistically significant after additional adjustment for psychotropic medication and psychiatric disorders. In contrast to the findings in the NK refugees, the GM volume ratios in both clusters were not significantly correlated with any clinical variables in the SK residents.

## Discussion

The present study demonstrated structural differences in thalamic nuclei between NK refugees and SK residents. Specifically, NK refugees exhibited greater thalamic GM volume ratios in the right medial-posterior nuclei and left medial nuclei compared with SK residents. Additionally, greater GM volume ratios in both clusters were associated with less severe depression in NK refugees, but not in SK residents.

Consistent with our hypothesis, GM volumes in specific thalamic nuclei differed between NK refugees and SK residents. Thalamic structure and function are reportedly affected by genetic (36) and environmental factors (37). The populations of NK and SK share an ethnic background, language, history, and culture. Exposure to trauma, which is much more common in NK refugees, may therefore play an important role in their thalamic alterations.

In the current study, NK refugees had increased GM volume in thalamic nuclei. Previous studies have suggested that increased thalamic volume is associated with resilience to traumatic experiences. An association between reduced thalamic volume and increased social avoidance has been observed in mice experiencing social defeat stress (38). Reduced thalamic volume is reportedly associated with the frequency and duration of re-experience of trauma in traumatized humans (39). Thalamic volume is also reportedly associated with childhood maltreatment among high school students and exposure to trauma among deployed soldiers (15, 40). While reduced thalamic volume is reportedly associated with psychological disturbances after trauma, increased thalamic GM volume might reflect neurobiological resilience to trauma.

Consistent with this notion of resilience, the current study found that NK refugees with fewer depressive symptoms tended to have increased thalamic GM volume. Many previous studies have also reported a relationship between depression and thalamus. Reduced volume and shape deformation have been reported in patients with depressive symptoms (9–11). Thalamic structural changes in patients with depressive symptoms were reported to recover after remission of depression (41). As brain structural changes have been reported to represent resilience to traumatic experiences by reducing psychiatric symptoms (26, 27, 42), increased thalamic GM volume is associated with fewer depressive symptoms in NK refugees.

In the present study, the NK refugees were more depressed, but had increased thalamic GM volume, compared with SK residents. The association was significant after controlling ICV, age, sex, and the number of traumatic experiences. This appears counterintuitive because greater depression in NK refugees was correlated with a “reduction” of thalamic GM volume. The increased thalamic GM volume in NK refugees (compared with SK residents) may either be a compensatory mechanism to prevent depression after trauma or a pre-existing factor protecting against depression before trauma. However, because this study used a cross-sectional design, the causal relationship between thalamic changes and trauma remains unclear. Because thalamic volume has been reported to predict the treatment response of patients with major depressive disorder (43), NK refugees with greater thalamic GM volume may be more resistant to the development or progression of depression compared with NK refugees, who have smaller thalamic GM volumes. Despite their higher depression levels, our NK refugees had greater thalamic GM volume than SK residents; therefore, a compensatory mechanism after trauma appears more plausible. The lack of a correlation between depression and thalamus in SK residents (in contrast to NK refugees) also supports the existence of a compensatory mechanism after trauma. Because thalamic structure has been reported to change after trauma (39), long-term and repeated trauma among NK refugees may induce the compensatory thalamic changes, while in non-traumatized SK residents, no such compensatory alteration is required.

In the present study, the NK refugees had increased thalamic GM volumes in the right medial-posterior nuclei and left medial nuclei. Posterior group includes the pulvinar and the geniculate nuclei, where the additional analysis showed increased GM volumes in the NK refugees. The pulvinar nucleus has been identified as a key structure for modulating both emotional and attentional processes (44, 45). The pulvinar nucleus is also considered important for fearful stimuli processing (5, 46). The recruitment of a subcortical pathway from the pulvinar nucleus to the amygdala facilitates fear recognition (47, 48). In particular, explicit fear processing elicits greater activation of the pulvinar nucleus, which suggests the importance of visual attention during explicit fear processing (49). Additionally, the medial geniculate nucleus has been identified as a critical component for fear conditioning (50, 51). It is also involved in emotional behavior by projecting to several subcortical areas (52). The lateral geniculate (53) and suprageniculate nuclei (54) have also been suggested to participate in fear processing. The lateral geniculate nucleus is involved in selective attention (55), whereas the suprageniculate nucleus is involved in emotional processing (56). Based on previous studies, we can speculate that the posterior nuclei play a crucial role in enhancing regulation of fear and emotion after trauma exposure. Consistent with previous findings regarding an association between depression and structural deficits in the posterior nuclei (57, 58), reduced GM volume in the right posterior nuclei in our traumatized NK refugees was associated with severer depressive symptoms.

The medial group includes the mediodorsal and the reuniens nuclei, where NK refugees showed greater GM volumes than SK residents, although the statistical significance is not enough for Bonferroni correction for multiple comparison. The mediodorsal nucleus plays a role in emotional processing and fear extinction through connections to limbic circuitry (59, 60). The reuniens nucleus, as a part of the medial group, also mediates contextual fear conditioning and promotes resilience to depression (61, 62). The current study revealed increased thalamic GM volumes in the bilateral medial nuclei of NK refugees. Additionally, the increased thalamic GM volumes in the bilateral medial nuclei were associated with decreased depression. The increased medial nuclei volumes might also reflect neurobiological resilience to trauma and serve as a compensatory mechanism for depression through modulation of emotion and fear after trauma.

To our knowledge, this is the first study to investigate thalamic GM volume changes in traumatized refugees. However, this study had several limitations. Various trauma characteristics (e.g., severity, duration, and time since trauma) were not evaluated in detail. The chronic and repetitive nature of the traumas experienced by refugees makes it difficult to assess these features. We attempted to address this limitation by counting the number of traumatic experiences to estimate trauma burden. Second, because the study used a cross-sectional design, causal relationships could not be determined. The retrospective nature of cross-sectional studies is also vulnerable to recall bias and decreases the reliability of the results. Longitudinal analysis may be needed to clarify specific causal relationships without recall bias. Third, handedness could not be explored, as handedness data were not present for some participants. Although some previous studies reported the absence of the associations between thalamic GM volumes and handedness (63, 64), handedness should be considered in future studies. Another limitation of the present study is that it included SK residents, rather than NK residents, as a control group. Generally, it is not possible to recruit people currently residing in North Korea, and non-traumatized NK refugees are very rare. Because SK residents share a similar genetic and historical background with NK residents, we considered SK residents to be the best alternative control group.

## Conclusion

In conclusion, our study demonstrated that NK refugees had increased thalamic GM volumes in right medial-posterior nuclei and left medial nuclei. Increased GM volumes in these nuclei were associated with reduced depression in NK refugees. These results suggest that the medial and posterior thalamic nuclei might play a role in neurobiological resilience after trauma through enhanced emotional regulation.

## Data Availability Statement

The original contributions presented in the study are included in the article, further inquiries can be directed to the corresponding author.

## Ethics Statement

The studies involving human participants were reviewed and approved by the Institutional Review Board of Seoul National University Hospital. The patients/participants provided their written informed consent to participate in this study. Written informed consent was obtained from the individual(s) for the publication of any potentially identifiable images or data included in this article.

## Author Contributions

JJ, SY, SL, YL, and SK: conceptualization. JiL, NK, and HJ: methodology and data curation. JoL, YL, and SK: validation and writing – review and editing. JiL and NK: formal analysis. SK: investigation and funding acquisition. JJ, SY, and SL: resources. JiL: writing – original draft preparation. NK: visualization. YL and SK: supervision. All authors have read and agreed to the published version of the manuscript.

## Conflict of Interest

The authors declare that the research was conducted in the absence of any commercial or financial relationships that could be construed as a potential conflict of interest.

## Publisher’s Note

All claims expressed in this article are solely those of the authors and do not necessarily represent those of their affiliated organizations, or those of the publisher, the editors and the reviewers. Any product that may be evaluated in this article, or claim that may be made by its manufacturer, is not guaranteed or endorsed by the publisher.

## Footnotes

1
www.fil.ion.ucl.ac.uk

2
http://marsbar.sourceforge.net
